# New-Onset Neurologic Symptoms and Related Neuro-Oncologic Lesions Discovered After COVID-19 Vaccination: Two Neurosurgical Cases and Review of Post-Vaccine Inflammatory Responses

**DOI:** 10.7759/cureus.15664

**Published:** 2021-06-15

**Authors:** Evan H Einstein, Andia Shahzadi, Likowsky Desir, Joshua Katz, John Boockvar, Randy D'Amico

**Affiliations:** 1 Neurosurgery, Lenox Hill Hospital/Donald and Barbara Zucker School of Medicine, New York, USA

**Keywords:** covid-19 vaccine, neurological symptoms, melanoma, glioma, inflammation

## Abstract

A global effort is underway to distribute coronavirus disease 2019 (COVID-19) vaccines to limit the crisis. Although adverse events related to vaccination are rare, there have been cases of new-onset neurologic symptoms following vaccination. We present two cases of new-onset neurologic symptoms post-vaccination that, upon further workup, revealed two different neuro-oncologic processes requiring neurosurgical intervention and further treatment. We hypothesize that despite these processes being unrelated to vaccination, the COVID-19 vaccines may induce an inflammatory cascade with the ability to uncover underlying sinister pathology. Our report therefore emphasizes the need for careful evaluation in the setting of new-onset neurologic symptoms after COVID-19 vaccination.

## Introduction

Currently, a global effort to distribute and administer coronavirus disease 2019 (COVID-19) vaccines is underway with more than 1.38 billion doses administered as of this writing [1]. Two vaccines in particular, Pfizer (COVID-19 mRNA vaccine BNT162b2) and Moderna (mRNA-1273 vaccine), have demonstrated over 90% efficacy with no safety threat [2]. However, vaccine rollout has not been without stringent caution or adverse events. Adverse events have included transient symptoms such as fever/chills, headaches, fatigue, myalgia/arthralgia, lymphadenopathy, nausea or local swelling, erythema, or pain that resolve spontaneously without permanent deficit [3]. More recently, distribution of Johnson & Johnson’s vaccine (As26.COV2.S) was paused in the United States over concern for cerebral venous sinus thrombosis (CVST) and thrombocytopenia after vaccine administration [4]. These effects may fuel vaccine hesitancy and threaten reduced vaccination rates. Many have called on medical professionals to reduce fears surrounding vaccination through transparent and effective communication to reduce the risk of COVID-19.

Cases of neurologic symptoms related to vaccination have been uncommon. To date, only one description of Guillain-Barre syndrome (GBS) after vaccination has been published [5]. Another report of a patient developing a Bell’s palsy after COVID-19 vaccination has been described, although a causal relationship remains unclear [6]. We describe two patients that presented with new neurologic deficits after the COVID-19 vaccination that subsequently revealed two different neuro-oncologic diagnoses. We hypothesize that a post-vaccine inflammatory response resulted in the hyperacute presentation of these lesions. These cases further emphasize the need to cautiously consider and evaluate new neurologic symptoms following COVID-19 vaccination.

## Case presentation

Case 1

**Figure 1:**
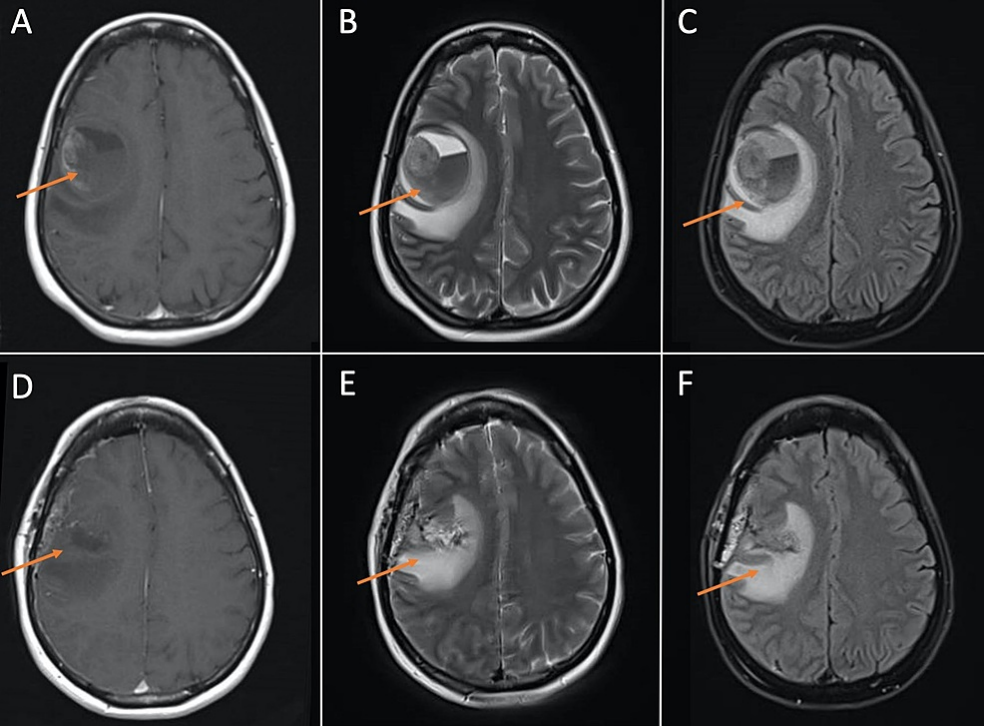
MRI brain demonstrating large hemorrhagic cavity in the right frontal lobe with an enhancing focus along the right superolateral margin representing a hemorrhagic mass. (A), (B), and (C) are pre-operative T1, T2, and FLAIR images, respectively. (D), (E), and (F) are post-operative T1, T2, and FLAIR images, respectively, demonstrating resection of mass and post-surgical changes. FLAIR: fluid-attenuated inversion recovery.

A 58-year-old woman with a remote history of an excised right arm melanoma with negative axillary lymph nodes presented to our hospital’s emergency department with slurred speech and worsening left facial droop over two weeks following her second dose of a COVID-19 vaccine. She had received no further chemotherapy or radiation after her resection eight years prior and had appropriate follow-up. Following the second dose of a COVID-19 vaccine, she developed high-grade fevers and malaise and new-onset left facial weakness. The patient’s symptoms were attributed to a post-vaccination Bell’s palsy and she was recommended no further workup. Fevers and malaise resolved spontaneously over the next 24-48 hours as is typical with post-vaccination symptoms, but the facial weakness persisted until approximately two weeks later when she developed acute worsening of her facial droop with associated slurred speech, drooling, and new-onset left arm and leg weakness. She was taken to the emergency department and underwent stroke evaluation including computed tomography (CT) of the head which demonstrated a 3.4 cm right intraparenchymal hemorrhage with a 3 mm midline shift. CT angiography of head and neck did not demonstrate any abnormalities. Complete blood count, comprehensive metabolic panel, and coagulation factors did not demonstrate any overt abnormalities. Contrast-enhanced MRI of the brain demonstrated a large hemorrhagic cavity in the right frontal lobe with an enhancing focus along the right superolateral margin representing a hemorrhagic mass (Figure 1). The patient was taken promptly to the operating room for hematoma evacuation and excisional biopsy of the enhancing lesion. Final pathology was consistent with a metastatic malignant melanoma, with immunohistochemical profile demonstrating positive staining for HMB-45, Melan-A, and S100. 

Case 2

**Figure 2:**
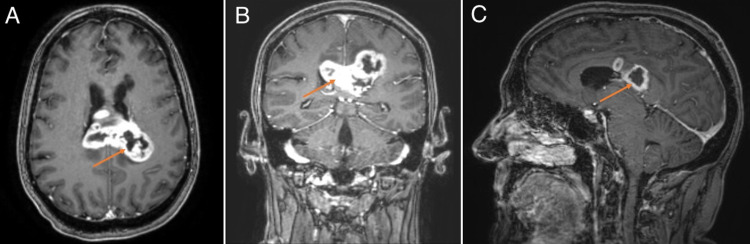
MRI brain demonstrating a heterogeneously enhancing mass with cystic and necrotic changes centered within the splenium of the corpus callosum. (A), (B), and (C) are T1-weighted post-contrast axial, coronal, and sagittal views, respectively.

A 52-year-old woman with a medical history of hypothyroidism and breast cancer presented after developing severe headache and neck stiffness associated with intermittent high-grade fevers to 102ºF four days after her first dose of a COVID-19 vaccine. Her primary care physician initially recommended monitoring of her symptoms and the use of over-the-counter analgesics which provided relief of her fevers. Her headaches persisted and the patient was prescribed a short course of methylprednisolone which reduced their severity. Symptoms returned two days after completion of the steroids and the patient was prescribed a second course of steroids and was sent for computed tomography (CT) of the chest and head which demonstrated a lesion arising within the corpus callosum. Contrast-enhanced MRI demonstrated a 5.8 cm heterogeneously enhancing mass with cystic and necrotic changes centered within the splenium of the corpus callosum (Figure 2). Pre-operative laboratory tests were overall unremarkable except for a mild anemia (hemoglobin 10.1 g/dL) and mild decreased calcium at 8.2 mg/dL. The patient underwent a biopsy which demonstrated an IDH-wildtype, World Health Organization Grade IV glioblastoma, with positive staining for glial fibrillary acidic protein (GFAP), negative staining for IDH-1, and rare positive staining for p53. The patient was ultimately transferred to our facility for definitive treatment. 

## Discussion

Vaccination against the COVID-19 virus has demonstrated its efficacy and importance to avoid an unnecessary prolongation of the pandemic. There is a need to educate the public on the safety of these vaccines and to provide effective communication to reduce hesitancy so patients can make informed decisions regarding the vaccine. It is critical to understand that the cases described above do not demonstrate adverse reactions to the COVID-19 vaccine, but instead call physicians to cautiously consider and evaluate new neurologic symptoms following COVID-19 vaccination. 

The pathogen, severe acute respiratory syndrome coronavirus 2 (SARS-CoV-2), targets cells through its viral structural spike protein [7]. While infection typically produces upper respiratory symptoms and often pneumonia, symptoms can progress to severe disease states including sepsis and acute respiratory failure necessitating intensive care in 5% of those overall harboring COVID-19 infections and up to 20% of those hospitalized with the disease [7]. Sequelae and complications related to COVID-19 are still being explored, but it is clear that multiple organs systems can be affected including the nervous system. To date, the most common neurological complaints associated with COVID-19 infection include anosmia, ageusia, headache, as well as more serious complications such as stroke, seizures, and encephalopathy [8]. Neurologic symptoms related to vaccination otherwise remain extremely rare [5,6].

We report two cases of new-onset neurological symptoms after the COVID-19 vaccination. In both cases, further diagnostic testing revealed neuro-oncologic processes that required neurosurgical intervention. Administration of these vaccines was unrelated to the oncologic diagnoses themselves. However, these two independent processes both came to the clinical forefront following vaccination. We hypothesize that the inflammatory response to the COVID vaccine may have played a role in increasing clinical symptoms in these patients, potentially in relation to the COVID-19 spike protein.

The mRNA-based vaccines specifically are designed to encode for the COVID-19 spike protein, and one particular report described 4 cases of delayed inflammatory reactions after hyaluronic dermal filler implantations in the setting of recent COVID-19 vaccination [9]. The authors hypothesized a potential mechanism of inflammation involving the spike protein and its typical binding site for blockade on angiotensin 2 converting enzyme receptors (ACE-2). Since the mRNA-based vaccines in particular are designed to encode for the spike protein itself, and there are a substantial number of dermal ACE-2 receptors, they hypothesized an inflammatory cascade was initiated in reaction to granulomas that had formed around residual hyaluronic acid particles in the skin.

Although the precise mechanism of post-vaccination inflammation is unknown, it is known that spike proteins can initiate inflammatory cascades and cross the blood-brain barrier (BBB) in COVID-19 infections [10,11]. It is possible that encoded spike proteins post-vaccination therefore cross the BBB and enhance inflammatory responses to nascent pathology within the brain following vaccine administration. We believe that an augmented inflammatory response following vaccination called attention to these neuro-oncologic diseases by exacerbating peritumoral edema and worsening clinical symptoms. 

## Conclusions

It remains critical to understand that the vaccine is not responsible for the cause of these underlying lesions. However, these cases emphasize the importance of carefully evaluating new-onset neurologic symptoms after COVID-19 vaccination, given the potential for the inflammatory response to uncover underlying pathology requiring treatment.
